# Ensuring older Australians remain socially connected during the COVID-19 isolation period

**DOI:** 10.1177/0004867420945780

**Published:** 2020-07-23

**Authors:** Andrew Page, Sandro Sperandei, Matthew J Spittal, Jane Pirkis

**Affiliations:** 1 Translational Health Research Institute, Western Sydney University, Penrith, NSW, Australia; 2 Melbourne School of Population and Global Health, The University of Melbourne, Parkville, VIC, Australia

To the Editor

The COVID-19 pandemic and subsequent policy responses in the Australian context have resulted in substantial changes to the way in which communities are currently functioning and potentially will have ongoing psychosocial impacts post-pandemic.

One of the key strategies to reduce the rate of infection has been physical distancing. Authorities have requested that people remain in their homes wherever possible and limit their travel to obtaining essential goods and services. This public health strategy is absolutely necessary and appears to be yielding the desired result in terms of ‘flattening the curve’. But it has required major adjustments for many and has been especially difficult for some. Older people are of particular concern. They have been strongly advised to distance themselves from family members and friends because they have higher case fatality. For them, the positive impacts of physical distancing may be accompanied by isolation and loneliness, which may in turn lead to significant psychological distress.

**Figure 1.**
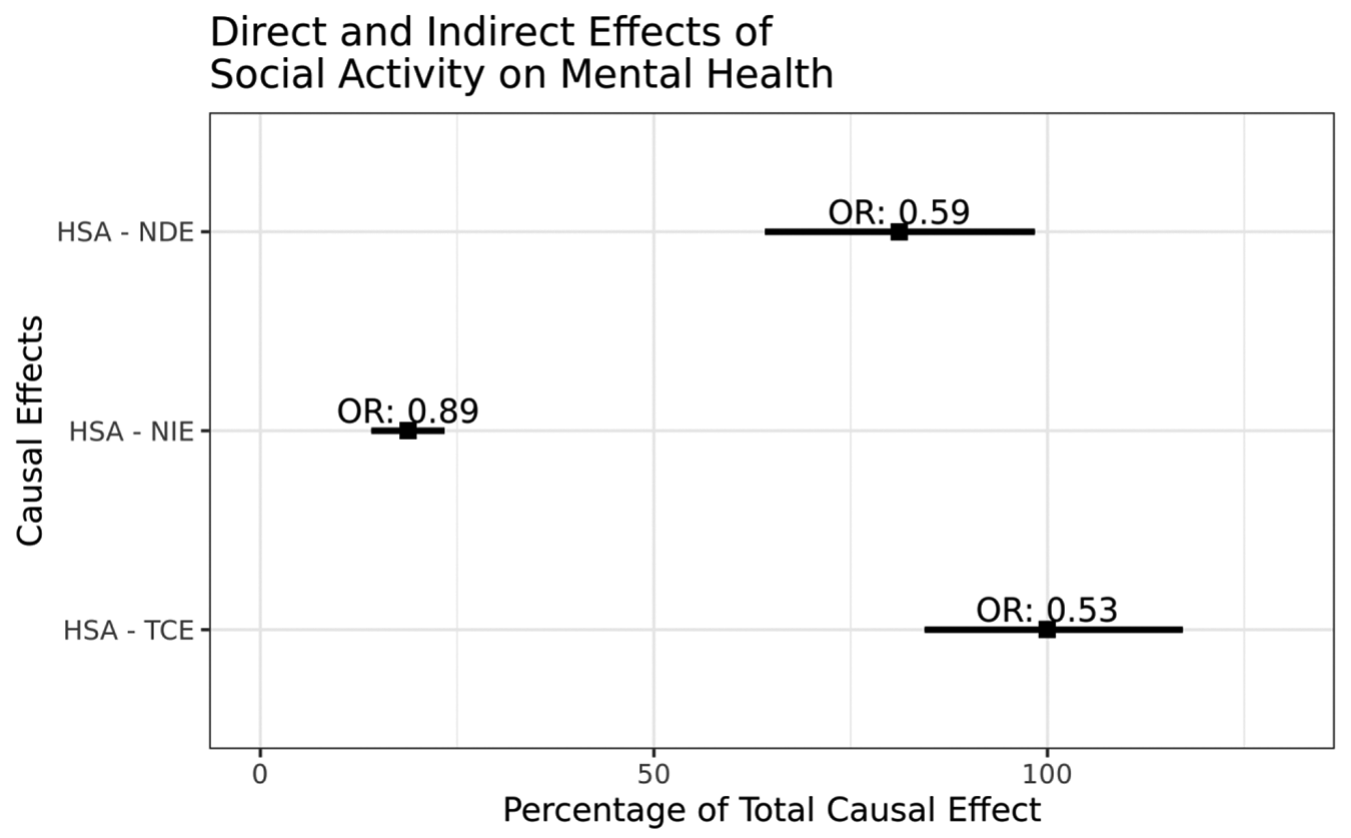
Natural direct effect (NDE) and natural indirect effect (NIE) of high social activities (HSA) on psychological distress as a percentage of the total causal effect (TCE). (Corresponding odds ratios [OR] and 95% confidence intervals are labelled.) Analyses were restricted to only those participants who (1) completed both the baseline and follow-up survey and (2) were classified as having ‘none’ or ‘mild’ psychological distress (K10 score <24) at baseline (*N* = 57,961). Mediation analysis using a counterfactual approach (Lange et al., 2012; VanderWeele, 2015) was used to assess the TCE of level of social activity (high/low) on psychological distress (K10 score ⩾24) (adjusting for sex, age group, educational achievement, migrant status, marital status, employment or retirement status, and household income). The TCE decomposed the association into (1) the NDE of physical social activity on psychological distress and (2) the NIE through non-physical social activities (talking with friends and family on the telephone) using the imputation-based approach.

We used the 45 and Up Study, a large prospective cohort study (*N* = 267,153) established in 2006 in New South Wales (Australia) with a focus on adults aged 45 years and older (45 and Up Study Collaborators, 2008), to investigate whether social connection (as measured by the Duke Social Support Index [DSSI]) was associated with lower levels of psychological distress among older age groups. The DSSI was classified into physical social activities (spending time with friends and family, and group social activities) and non-physical social activities (talking with friends and family on the telephone). A causal mediation analysis (Lange et al., 2012; VanderWeele, 2015) showed a strong association between higher level of social activity and low psychological distress (odds ratio [OR] = 0.53, 95% confidence interval [CI], 0.47, 0.58), and importantly that 19% of this protective association was caused through contact with friends and family by telephone (Figure 1). This finding suggests that even among those with lower physical social activity, having regular telephone contact can contribute to lower levels of psychological distress.

We absolutely agree that there is a need for everyone to maintain physical distance in these unprecedented times. We must ensure that this public health measure does not come at too great a cost – however, particularly for older people who find themselves alone and craving human contact. Maintaining social connections with this generation is crucial, and people have found that meeting from a distance, for example, across windows or doors, can be a way to continue social connection. Doing so by telephone may be a good temporary replacement for face-to-face visits, particularly for those who are not comfortable with online forms of communication.
